# Documented Readmission for Undernutrition and Associated Factors Among Children Under Five Years of Age at a Rural District Hospital in South Africa

**DOI:** 10.3390/nu18213541

**Published:** 2026-10-27

**Authors:** Lister Pitso, Phumudzo Mamphwe Tshiambara, Pedro Pisa

**Affiliations:** Department of Human Nutrition, Faculty of Health Sciences, University of Pretoria, Pretoria 0001, South Africa; u18150978@tuks.co.za or listermakhubs@gmail.com (L.P.); pedro.pisa@up.ac.za (P.P.)

**Keywords:** undernutrition, readmission, children under five, post-discharge follow-up, South Africa

## Abstract

Background/Objectives: Undernutrition remains an important contributor to morbidity and mortality among children under five years of age, particularly in rural and low-resource settings. This study determined the proportion of children with documented readmission for undernutrition and identified associated factors at Bela-Bela District Hospital, Limpopo Province, South Africa. Methods: A retrospective analytical hospital record review covering January 2018 to December 2025 was conducted. Of 538 medical records identified, 94 were excluded, 444 remained eligible, and 222 were selected through systematic random sampling. Clinical, caregiver/household conditions, healthcare-related and post-discharge data were extracted. The primary outcome was documented readmission for undernutrition at Bela-Bela District Hospital following discharge. Multivariable logistic regression was used to identify independently associated factors. Results: Of 222 children, 54 (24.3%) had documented readmission. Previous admission for undernutrition was strongly associated with documented readmission (AOR = 4.12; 95% CI: 2.00–8.50; *p* < 0.001). Lack of documented caregiver nutrition education (AOR = 3.10; 95% CI: 1.40–6.90; *p* = 0.005), absence of a documented follow-up date (AOR = 2.20; 95% CI: 1.05–4.60; *p* = 0.036), and failure to attend follow-up according to schedule (AOR = 1.95; 95% CI: 1.05–3.60; *p* = 0.030) were also associated with documented readmission. Conclusions: Documented readmission was associated with previous admission for undernutrition and gaps in documented post-discharge care. These findings highlight the importance of strengthening post-discharge planning, documentation of caregiver nutrition education, documentation of follow-up appointments, and continuity of nutritional support.

## 1. Introduction

Undernutrition remains a major public health challenge and a leading nutrition-related contributor to illness and death among children under five years of age, particularly in low- and middle-income countries. Undernutrition contributes to approximately 45% of deaths in this age group, and continues to place pressure on households and health systems [1,2,3,4]. Although the global prevalence of undernutrition has declined, mortality among affected children remains substantial, with reported rates ranging from 0.8% to 10.3% among children treated for severe acute malnutrition [1,2,5,6].

Undernutrition presents as underweight, stunting, and wasting. Wasting is the most acute form and is strongly associated with complications and an increased risk of mortality. Globally, about 47 million children under five years of age are affected by wasting [2,6]. Acute undernutrition is commonly defined by low weight-for-height and is classified as either moderate or severe acute undernutrition [2,5,7,8]. It is most common between 6 and 18 months of age, a period marked by rapid growth, high nutritional demand, and increased vulnerability to inadequate dietary intake and infection [2]. The present study focused on acute forms of undernutrition identified through weight-for-height Z-scores, mid-upper arm circumference, and the presence of bilateral oedema. However, the term undernutrition was retained because it was the diagnostic terminology used within the reviewed hospital records.

In 2022, the global prevalence of acute undernutrition was estimated at 6.8%, while Africa reported a prevalence of 5.8% [7]. South Africa reported a lower prevalence than many other African countries, estimated at 3.4% [9]. However, undernutrition remains a concern within the South African context, with Limpopo Province among the provinces most affected, reporting high levels of underweight (69%) and wasting (11.1%) among children under five years of age [1,10,11,12,13].

The consequences of undernutrition extend beyond short-term clinical recovery. Undernutrition is associated with impaired physical growth, delayed cognitive development, increased susceptibility to infection, and greater household and health-system burdens. Repeated episodes may worsen these effects. Anthropometric recovery after treatment does not always indicate full biological recovery, as children remain vulnerable to relapse and infection after discharge [2,10]. Studies have reported that some children treated successfully for acute undernutrition fail to sustain recovery, with readmissions occurring within 3 to 6 months after discharge in some settings [2,3]. Reported relapse rates vary widely, ranging from 0% to 65%, with post-recovery mortality reported up to 10.4% within 12 months in some studies [2,3].

Relapse following treatment may be defined differently across studies, with some studies defining it as a return to moderate or severe acute malnutrition after a documented nutrition recovery, while routine hospital studies may identify relapse through a subsequent admission for undernutrition. Undernutrition treatment is influenced by clinical, household, caregiver, and health-system factors. Previous undernutrition, poor socioeconomic conditions, limited access to healthcare, recurrent illness, poor anthropometric recovery, inadequate caregiver nutrition knowledge, and weak follow-up systems have been associated with relapse in previous studies [1,7,13,14,15]. Programme-related factors are especially important because treatment success depends not only on inpatient stabilisation but also on sustained recovery after discharge. Gaps in nutrition education, food supplementation, follow-up appointments, and adherence to scheduled visits may increase the risk of relapse among vulnerable children.

Despite improvement in the identification and treatment of childhood undernutrition, limited evidence is available on readmission rates after hospital discharge, particularly in rural South African settings. Most available studies focus on the prevalence, inpatient management, or the immediate treatment outcomes, with less attention given to whether children sustain nutrition recovery after returning to their households. Evidence on documented readmission and the clinical, caregiver, and health-system factors associated with it may support improved discharge planning and post-discharge care. Therefore, this study determined the proportion of children with documented readmission for undernutrition and identified associated factors among children under five years of age treated at Bela-Bela District Hospital in Limpopo Province, South Africa, between January 2018 and December 2025.

## 2. Materials and Methods

### 2.1. Study Design and Setting

A retrospective analytical hospital record review was conducted in the paediatric unit of Bela-Bela District Hospital, Limpopo Province, South Africa. Routinely collected hospital records of children admitted with moderate or severe acute undernutrition between January 2018 and December 2025 were reviewed. The study examined clinical, anthropometric, caregiver, household, nutrition-services, and follow-up information documented during routine hospital care. Bela-Bela District Hospital serves predominantly rural communities, supported by four fixed clinics and two mobile clinics. The area experiences socioeconomic challenges, including unemployment, poverty, and limited access to healthcare services, which may affect child nutrition, treatment adherence, and post-discharge follow-up [16,17,18,19].

### 2.2. Study Population

The study population comprised medical records of children under five years of age admitted to the paediatric unit of Bela-Bela District Hospital with moderate or severe undernutrition between January 2018 and December 2025. For each child, the index admission was defined as the first qualifying undernutrition admission selected for analysis during the study period. A history of undernutrition admission referred to a documented admission for undernutrition occurring before the index admission, while documented readmission for undernutrition referred to a subsequent admission occurring after discharge from the index admission at Bela-Bela District Hospital during the study period. These were treated as separate variables to distinguish previous admission history from the study.

A total of 538 medical records of children under five years of age admitted with moderate or severe undernutrition between January 2018 and December 2025 were identified. Records were excluded if the child was transferred to another facility before completing treatment; if data were missing, including the discharge summary and other relevant information required for the study (dietitian notes and admission notes); or if the child was diagnosed with a congenital disorder.

Children were eligible if they were under 5 years of age at their index admission for moderate or severe undernutrition at Bela-Bela District Hospital. Subsequent admission for undernutrition was included in the outcome assessment only if the child remained under five years of age (before celebrating their fifth birthday) at the time of admission. An admission occurring after the child’s fifth birthday was not included on the study outcome assessment, but the qualifying index admission remained eligible for the study.

Of the 538 medical records identified during the study period, 94 were excluded: records for children transferred out to another health care facility (30) before completing treatment; records without a discharge summary and other relevant information (55); medical records for children diagnosed with congenital disorders (9). A total of 444 records remained eligible. For all the children included in the study, all subsequent admissions were recorded as a documented readmission outcome, but only if the child remained under five at the time of their subsequent admission.

### 2.3. Sampling Procedure and Eligibility Criteria

During protocol development, a minimum sample requirement of 174 records was estimated using α = 0.05, 80% power, an anticipated odds ratio of 2.0, and an expected readmission proportion of approximately 25%. A total of 538 medical records of children admitted with undernutrition at Bela-Bela District Hospital between January 2018 and December 2025 were identified. Following the application of the inclusion and exclusion criteria, 94 records were excluded, leaving 444 eligible records.

Systematic random sampling was applied across the eligible record list until 222 records were selected from the 444 eligible records, exceeding the minimum sample requirement of 174.

The total number of eligible records was divided by the required sample size to determine the sampling interval (444/222 = 2). A starting point was randomly selected between 1 and 2, resulting in record number 2 being chosen as the starting point, and every second eligible record was included until the total of 222 records was reached. The final sample included 222 eligible records chosen for analysis. The remaining 222 records from the eligible record list were not selected for analysis. For each selected medical record, the first admission during the study period was considered an index admission and all the predictor information was obtained from that admission. The predictor information included demographic, clinical, anthropometric, caregiver, household, and healthcare characteristics. Any documented admission for undernutrition before the index admission was recorded as previously admitted for undernutrition.

A subsequent admission for undernutrition at Bela-Bela District Hospital during the study period following treatment and discharge was classified as an outcome. The age criterion was applied at the index admission; children under 5 years of age at index admission were eligible for the study. A subsequent admission was included in the outcome assessment only if the child remained younger than five years of age at the time of that admission, though the child’s index admission remained eligible for inclusion in the study. This study only included children under five years of age admitted with moderate or severe undernutrition to the paediatric unit in Bela-Bela District Hospital between January 2018 and December 2025. Follow-up records were reviewed for subsequent undernutrition admissions occurring up to six months after discharge from the index admission. The outcome was analysed as a binary six-month documented readmission outcome rather than as person-time or time-to-event data. No non-admitted comparison group was included or analysed.

### 2.4. Data Collection

Data were collected from the medical records using a structured data extraction instrument that was developed by the researcher in accordance with the study objectives, existing hospital and dietetic documentation, previous studies, and relevant WHO/UNICEF guidance [4,8,20,21]. The instrument covered child, anthropometric, clinical, feeding, caregiver, household, supplementation, counselling, and follow-up variables. Before full extraction, the instrument was piloted on five records to assess its clarity, completeness, and usability; however, it was not formally psychometrically validated. Data extraction was undertaken by the researcher and the extracted information checked by a trained dietitian for omissions, duplicate entries, and transcription inconsistencies.

Although the data extraction instrument contained fields for anthropometric measurements at discharge, these measurements were not consistently documented in the available medical records and were therefore excluded from the final analysis of the predictor variables. This study was a retrospective observational record review, and no study-specific treatment programme was introduced. Children received routine inpatient management for undernutrition according to the existing hospital practices. Routine discharge care included nutritional supplementation, caregiver nutrition counselling, and arrangement of post-discharge follow-up; these components were abstracted as documented in the records.

In routine clinical practice, children discharged following treatment for undernutrition continued receiving nutrition follow-up until their weight-for-height measurements improved to greater than −1 SD. This clinical follow-up was not restricted to a fixed six-month period. However, for the purpose of this retrospective study, documented undernutrition-related readmissions were assessed within six months following discharge from the index admissions. Information was extracted from available hospital records, and no additional prospective follow-ups were conducted.

### 2.5. Variables

Outcome Variable: The primary outcome was a documented subsequent admission for undernutrition at Bela-Bela District Hospital after discharge from the index admission. The outcome was classified as either “documented readmission” or “no documented readmission” based on available medical records. The study protocol specified a six-month readmission assessment window following index discharge. The first admission during the study period was recorded as an index admission. The subsequent admission was documented as a readmission only when the child was still under five years of age during that admission. Admissions occurring at other healthcare facilities were not routinely captured. Only subsequent admissions occurring while the child was under five years of age were included in the outcome assessment. Children without a documented readmission were not necessarily considered to have achieved nutritional recovery.

Moderate acute undernutrition was identified using the documented clinical diagnosis and anthropometric measurements, including a weight-for-height Z-score (WHZ) of at least −3 and below −2 standard deviations, and/or a mid-upper arm circumference (MUAC) of 11.5–12.4 cm in the absence of bilateral oedema. Severe acute undernutrition was identified from the documented clinical classification, including a weight-for-height Z-score below −3 standard deviations; and/or a mid-upper arm circumference below 11.5 cm; and/or the presence of bilateral pitting oedema.

Independent Variables: The independent variables were grouped into child clinical factors, feeding practices, caregiver and household characteristics, and health-system factors. Child clinical factors included age, sex, history of undernutrition admission, weight-for-height Z-score, mid-upper arm circumference, oedema, HIV status, and diarrhoeal illness.

For this multivariable model, nutritional severity was collapsed using anthropometric criteria only. Severe nutrition status was defined as a WHZ below −3 SDs and/or an MUAC < 11.5 cm, while oedema was added separately.

Caregiver and household variables included caregiver education level, employment status, and the available household socioeconomic characteristics. Health-system variables included documented nutritional supplementation, caregiver nutrition education, provision of a post-discharge follow-up date, and attendance according to the documented follow-up schedule.

For the multivariable analysis, caregiver education was categorised as lower education (no formal education or primary education) and secondary or higher education (secondary or tertiary education). Secondary or higher education was used as the reference category. Records with missing caregiver education information were excluded from the complete-case adjusted model.

Documented caregiver nutrition education referred to whether caregiver education was recorded in the available hospital or dietetic records. Absence of documentation was not interpreted as definitive evidence that counselling had not occurred.

A documented follow-up date referred to a recorded post-discharge appointment date in the available hospital records at Bela-Bela District Hospital during the study period. Documented attendance according to the follow-up schedule referred to recorded attendance at the scheduled follow-up appointment. This was a documentation-based follow-up measure, and absence of documented attendance was not interpreted as a confirmed default, because routine records did not always distinguish between no appointment, an appointment not yet due, attendance elsewhere, or true non-attendance. No documented attendance according to the schedule included absence of documented attendance at one or more scheduled appointments on the medical records. This definition was applied to children with and without a documented readmission for undernutrition. Children admitted before their scheduled follow-up appointment were not classified as failing to attend their scheduled appointment. These variables were based on the information that was available in the medical records.

### 2.6. Data Analysis

Data were captured and analysed using SPSS version 30.0. Descriptive statistics and analysis, including frequencies and percentages, were used to describe child, clinical, feeding, caregiver, household, and health-system characteristics. The proportion with documented readmission was calculated by dividing the number of children with a documented subsequent admission for undernutrition by the total number of records reviewed. Univariable analyses were initially conducted to explore relationships between the study variables and documented readmission. Multivariable logistic regression was then performed to identify factors independently associated with documented readmission after adjustment for selected clinical, caregiver, and health-system variables. Variables were included based on the initial analysis and their conceptual relevance to documented readmission for undernutrition. Adjusted odds ratios, 95% confidence intervals, and the *p*-values were reported. A *p*-value below 0.05 was considered statistically significant. Categories were collapsed to support model stability.

Missing information was retained as missing and not automatically interpreted as absence of the characteristics. Sixteen records (7.2%) lacked caregiver education level and were excluded from the complete-case multivariable analysis. Complete-case analysis for the final multivariable model, therefore, included 206 children, comprising 49 documented readmission events and 157 non-events. Eight predictor parameters were included in the final adjusted model, corresponding to approximately 6.1 outcome events per predictor parameter; adjusted estimates were therefore interpreted cautiously because of possible overfitting.

### 2.7. Ethical Considerations

Ethical approval was obtained from the University of Pretoria, Faculty of Health Sciences Research Ethics Committee, reference number: 326/2025-Line 1, with renewal approval obtained. Research permissions to access and review the medical records were also obtained from the Limpopo Department of Health, Ref: LP_2025-07-007, and Bela-Bela District Hospital before data collection commenced. Patient confidentiality was maintained throughout the study. Patient names and other identifying information were not captured in the dataset. Each record was assigned a unique study code for data collection and analysis. Data were stored securely and used only for research purposes.

## 3. Results

Of the 222 children included in the study, fifty-four of the 222 children (24.3%) had a documented subsequent admission for undernutrition at the study hospital. The remaining 168 (75.7%) had no subsequent admission documented at Bela-Bela District Hospital during the observation period.

### 3.1. Description of the Study Population

A total of 222 children were included in the descriptive analysis. The largest age group was children aged 6–24 months old (91/222; 41.0%), followed by 25–48 months old (78/222; 35.1%). Females accounted for 133 cases (59.9%), and males 89 cases (40.1%). A history of undernutrition admission was documented in 62 children (27.9%). Regarding anthropometric characteristics, a WHZ below −3 SDs was recorded in 107 children (48.2%), while 95 (42.8%) had a WHZ between −3 and <−2 SDs; in addition, an MUAC of 11.5–12.4 cm in 77 cases (34.7%) and <11.5 cm in 68 cases (30.6%) was recorded. Any grade of bilateral pitting oedema was documented in 125 cases (56.3%).

Most caregivers were unemployed (196/222; 88.3%), and many children lived in rural households (176/222; 79.3%). Child support grants were the most frequently documented household source of income (140/222; 63.1%). Nutritional supplementation was documented for 173 children (77.9%); caregiver nutrition education for 203 children (91.4%); a follow-up date for 175 children (78.8%); and attendance according to the documented follow-up schedule for 140 children (63.1%). Information on caregiver education level was missing for 16 children (7.2%). Clinical, anthropometric, demographic, caregiver, household, and health-system characteristics of the study population are presented in Table 1.

### 3.2. Readmission Proportion

Fifty-four of the 222 children (24.3%) had a documented subsequent admission for undernutrition at Bela-Bela District Hospital during the study period; the remaining 168 (75.7%) had no such admission recorded at the study hospital during the observation period. Documented readmission was similar between males and females. Among males, 22 of 89 (24.7%) had documented readmission for undernutrition compared to 32 of 133 females (24.1%). Thus, although females constituted a larger proportion of the hospital sample, there was no evidence that the predominance of females translated into a greater proportion of documented readmission.

### 3.3. Factors Associated with a Documented Readmission of Undernutrition

The complete-case adjusted model included 206 children, comprising 49 documented readmission events and 157 without documented readmission. Sixteen records with missing caregiver education-level information were excluded from the complete-case analysis. Among children with a history of previous undernutrition admission, 28 of 62 (45.2%) had a subsequent documented readmission compared with 26 of 160 (16.3%) among children without such a history. In the multivariable regression analysis, previous admission remained the strongest factor associated with documented readmission in the adjusted model (AOR = 4.12; 95% CI: 2.00–8.50; *p* < 0.001).

Absence of documented caregiver nutrition education was associated with increased adjusted odds of readmission (AOR = 3.10; 95% CI: 1.40–6.90; *p* = 0.005). Absence of a documented follow-up date was also associated with increased odds (AOR = 2.20; 95% CI: 1.05–4.60; *p* = 0.036), as was absence of documented attendance according to the follow-up schedule (AOR = 1.95; 95% CI: 1.05–3.60; *p* = 0.030). Severe nutritional status (AOR = 1.80; *p* = 0.072), caregiver education (AOR = 1.70; *p* = 0.058), oedema (AOR = 1.20; *p* = 0.500), and caregiver unemployment (AOR = 1.80; *p* = 0.120) were not statistically significant at the 0.05 level. The adjusted associations are presented in Table 2.

## 4. Discussion

In this retrospective hospital record review, 24.3% of children included in the study had a documented subsequent admission for undernutrition during the study period. In the adjusted model, history of undernutrition admission before index admission, lack of documented caregiver nutrition education, absence of a documented follow-up date, and lack of documented attendance category were associated with documented readmission. However, these events should be interpreted as documented hospital readmission rather than confirmed nutritional relapse, because nutritional recovery before each subsequent admission could not be consistently established.

The proportion observed in this study falls within the wide range of readmission estimates reported in other settings. However, direct comparison should be made cautiously as previous studies have differed in their study population, definitions of recovery and relapse, length of follow-up, treatment models, and methods used to identify subsequent episodes. Some studies actively followed children after documented recovery, whereas the present study relied on routine hospital records to identify subsequent readmissions [2,3,4,15,22].

Unlike studies that investigated children treated for severe acute undernutrition, this review included children admitted with both moderate and severe acute undernutrition. The 168 children (75.7%) without documented readmission should not be regarded as having achieved nutritional recovery; the study did not measure all treatment outcomes, and admissions to other hospitals or visits to primary health facilities may not have been captured. Therefore, the absence of documented readmission does not necessarily indicate sustained nutritional recovery.

Children with a documented history of undernutrition admission before the index admission had approximately four times the adjusted odds of a subsequent documented admission for undernutrition (AOR = 4.12; 95% CI: 2.00–8.50; *p* < 0.001) compared with those without such history. This association does not reflect the study eligibility criteria, as all included children had an index admission for undernutrition. It suggests that children with a history of earlier undernutrition admission may represent a group with increased vulnerability to subsequent readmission. This finding is consistent with studies showing that children treated for acute undernutrition remain vulnerable after discharge, even when anthropometric recovery has been achieved [3,4,23]. A documented history of undernutrition admission may therefore serve as a practical indicator for identifying children who could benefit from enhanced discharge planning, closer post-discharge monitoring, and improved linkage to primary health care and community-based nutritional services [7,24,25,26].

Absence of documented caregiver nutrition education was also associated with higher adjusted odds of documented readmission (AOR = 3.10; 95% CI: 1.40–6.90; *p* = 0.005). This finding highlights the potential importance of caregiver nutrition education in post-discharge care. Nutrition education may support caregivers in providing adequate nutritional intake, maintaining hygiene, recognising early signs of nutritional deterioration, and seeking timely healthcare [27,28]. However, the absence of documented caregiver nutrition education does not prove that nutrition education was not provided. Additionally, the retrospective nature of the study did not allow for assessment of the content, quality, duration, or caregiver understanding of the counselling provided. The observed association may therefore reflect differences in both service delivery and clinical documentation.

Similarly, an absence of a documented follow-up date and absence of attendance according to the recorded schedule had increased odds of documented readmission (AOR = 2.2; 95% CI: 1.05–4.60; *p* = 0.036 and AOR = 1.95; 95% CI: 1.05–3.60; *p* = 0.030). Post-discharge follow-up visits provide opportunities to monitor growth, reassess feeding practices, identify illness, reinforce caregiver counselling, and intervene before deterioration requires hospital readmission [2,4,27]. Poor follow-up may delay detection of early nutritional decline and increase the likelihood of relapse. In this study, failure to attend according to schedule referred to the absence of documented attendance after the scheduled appointment had passed. However, the retrospective study design could not allow the reasons for missed appointments to be established, including whether the appointment was missed due to transport costs, caregiver unavailability, migration, communication challenges, or attendance at another health facility.

In the adjusted model, a history of undernutrition admission before the index admission, absence of documented caregiver nutrition education, absence of a documented follow-up date, and no documented attendance according to the follow-up schedule were statistically associated with documented readmission. Nutritional severity (AOR = 1.80; *p* = 0.072), presence of oedema (AOR = 1.20; *p* = 0.500), and lower caregiver education (AOR = 1.70; 0.058) did not meet the prescribed 0.05 significance threshold. Other variables, including sex, feeding practices, supplementation, and broader household characteristics, were examined descriptively or in adjusted analyses but were not included in the final multivariable model. Therefore, no conclusions about their independent adjusted associations were drawn.

The absence of statistical significance should not be interpreted as though these factors are clinically not important. Previous findings suggest that children with severe anthropometric deficits appeared more likely to be readmitted than those with moderate deficits [29,30,31,32]. This finding should be interpreted with caution, as clinical severity remains clinically important because a low weight-for-height Z-score, a low mid-upper arm circumference, and oedema indicate a likelihood of physiological vulnerability and increased risk of complications [15]. Due to the retrospective nature of the study, routine records may not have captured the quality, quantity, duration, or adherence to feeding and supplementation. Limited variation in some household characteristics may also have reduced the ability to identify differences within this predominantly rural and socioeconomically disadvantaged population.

Many caregivers (88.3%) were not employed, and most children lived in rural households (79.3%). Child support grant (63.1%) was the most frequently reported source of household income. The absence of significance does not mean that these factors are unimportant. Some variables may have been affected by incomplete documentation, small category sizes, or limited variability within the study population.

Although females constituted a larger portion of the study population, 59.9% of the proportion with documented readmission for undernutrition was similar among females (32/133; 24.1%) and males (22/89; 24.7%). The higher representation of female children did not correspond to a higher observed proportion of readmission among females. The reason for female dominance in the study could not be established from the available records. Sampling was not stratified by sex, and the female predominance is therefore interpreted as a characteristic of the hospital case mix rather than evidence of a sex-specific readmission effect.

These findings support the importance of extending the management of childhood undernutrition beyond inpatient treatment. Children with a documented history of undernutrition admission may benefit from enhanced follow-up and closer nutritional monitoring. Strengthening post-discharge care through structured follow-up appointments, caregiver nutrition education, routine growth monitoring, assessment of household food insecurity, and referral to appropriate community-based support services may help address gaps in care. Establishing systems to identify and follow up with the parents of children who miss scheduled appointments could further improve continuity of care. A multidisciplinary approach involving doctors, nurses, dietitians, primary health providers, community health workers, and social workers may help facilitate coordinated nutritional support after hospital discharge. Although the effectiveness of these interventions was not evaluated in this study, they present potential strategies for addressing gaps in post-discharge care identified through the hospital record review.

## 5. Strengths and Limitations

This study has several strengths: First, it addresses a relatively understudied post-discharge outcome in a rural South African Hospital. Second, it draws on routinely collected records covering eight years (January 2018 to December 2025) to examine demographic, clinical, anthropometric, household, caregiver, and health-system characteristics. Third, the inclusion of both moderate and severe undernutrition provides information across different levels of nutritional severity.

However, several limitations were observed in this study: First, the retrospective study design depended on the completeness and accuracy of the routinely collected hospital records. Discharge anthropometric measurements and participant-level follow-up information were not consistently documented, limiting the ability to confirm nutritional recovery before subsequent admission and determine the available observation duration for all children. Second, Children with shorter observable follow-up may have had fewer opportunities for readmission to be documented. The reported 24.3% should therefore be interpreted as the observed proportion with documented readmission at the study hospital rather than a fixed-period cumulative incidence estimate. Third, readmissions occurring at other facilities were not captured, potentially underestimating recurrent admissions. Fourth, the complete-case adjusted model included 49 events for eight predictor parameters (approximately 6.1 events per parameter). Although categories were collapsed to reduce sparse cells, overfitting remains a concern, and adjusted estimates require cautious interpretation.

## 6. Conclusions

Among the children included in the study, 24.3% had a subsequent admission for undernutrition documented at Bela-Bela District Hospital during the study period. A history of undernutrition admission before the index admission showed the strongest adjusted association with documented readmission. Absence of documented caregiver nutrition education, absence of a documented follow-up date, and no documented attendance according to the follow-up schedule were also associated with increased adjusted odds. These findings support strengthening discharge planning, consistent documentation, and continuity of post-discharge nutritional care, particularly for children with previous admission.

The findings represent documented readmission at the study hospital rather than all episodes of recurrent undernutrition in the community, because complete nutritional recovery could not be established before subsequent admission. Prospective research using a standardised follow-up period is needed to further investigate recurrent undernutrition and evaluate strategies to improve post-discharge outcomes.

## Figures and Tables

**Table 1:** Child, clinical, feeding, caregiver, household, and health-system characteristics by documented readmission status.

Variables	Category	Total Population n = 222	Documented Readmission n = 54	No Documented Readmission n = 168
Child clinical factors				
Age (months)	0–5	15 (6.8%)	3 (5.6%)	12 (7.1%)
	6–24	91 (41.0%)	21 (38.9%)	70 (41.7%)
	25–48	78 (35.1%)	20 (37.0%)	58 (34.5%)
	49–59	38 (17.1%)	10 (18.5%)	28 (16.7%)
Sex	Male	89 (40.1%)	22/89 (24.7%)	67/89 (75.3)
	Female	133 (59.9%)	32/133 (24.1%)	101/133 (75.9)
History of undernutrition admission	Yes	62 (27.9%)	28/62 (45.2%)	34/62 (54.8)
	No	160 (72.1%)	26/160 (16.3%)	134/160 (83.8)
Weight-for-height Z-score	≥−1 SD	6 (2.7%)	2 (3.7%)	4 (2.4%)
	−2 to <−1 SDs	14 (6.3%)	3 (5.6%)	11 (6.5%)
	−3 to <−2 SDs	95 (42.8%)	19 (35.2%)	76 (45.2%)
	<−3 SDs	107 (48.2%)	30 (55.6%)	77 (45.8%)
Mid-upper arm circumference	≥13.5 cm	27 (12.2%)	6 (11.1%)	21 (12.5%)
	12.5–13.4 cm	46 (20.7%)	8 (14.8%)	38 (22.6%)
	11.5–12.4 cm	77 (34.7%)	20 (37.0%)	57 (33.9%)
	<11.5 cm	68 (30.6%)	18 (33.3%)	50 (29.8%)
	No data	4 (1.8%)	2 (3.7%)	2 (1.2%)
Oedema	No oedema	97 (43.7%)	23 (42.6%)	74 (44.0%)
	Oedema	125 (56.3%)	31 (57.4%)	94 (56.0%)
HIV	Exposed negative	38 (17.1%)	10 (18.5%)	28 (16.7%)
	Exposed positive	25 (11.3%)	6 (11.1%)	19 (11.3%)
	Unexposed negative	85 (38.3%)	20 (37.0%)	65 (38.7%)
	Unexposed positive	37 (16.7%)	8 (14.8%)	29 (17.3%)
	Unknown	37 (16.7%)	10 (18.5%)	27 (16.1%)
Diarrhoea	Yes	76 (34.2%)	21 (38.9%)	55 (32.7%)
	No	146 (65.8%)	33 (61.1%)	113 (67.3%)
Nutrition practices				
Exclusive breastfeeding	Yes	59 (26.6%)	12 (22.2%)	47 (28.0%)
	No	147 (66.2%)	38 (70.4%)	109 (64.9%)
	No data	16 (7.2%)	4 (7.4%)	12 (7.1%)
Complementary feeding initiation	<6 months	51 (23.0%)	10 (4.5%)	41 (18.5%)
	At 6 months	85 (38.3%)	22 (9.9%)	63 (28.4%)
	>6 months	70 (31.5%)	18 (%)	52 (23.4%)
	No data	16 (7.2%)	4 (1.8%)	12 (5.4%)
Caregiver characteristics				
Caregiver education	Lower	45 (20.3%)	14 (25.9%)	31 (18.5%)
	Secondary	131 (59.0%)	30 (55.6%)	101 (60.1%)
	Tertiary	30 (13.5%)	5 (9.3%)	25 (14.9%)
	No data	16 (7.2%)	5 (9.3%)	11 (6.5%)
Employment status	Employed	26 (11.7%)	5 (9.3%)	21 (12.5%)
	Unemployed	196 (88.3%)	49 (90.7%)	147 (87.5%)
Socioeconomic information				
Family size	<5	52 (23.4%)	12 (22.2%)	40 (23.8%)
	6–10	123 (55.4%)	30 (55.6%)	93 (55.4%)
	>10	33 (14.9%)	8 (14.8%)	25 (14.9%)
	No data	14 (6.3%)	4 (7.4%)	10 (6.0%)
Children under 5 years of age in household	≤2	94 (42.3%)	20 (37.0%)	74 (44.0%)
	3–5	94 (42.3%)	25 (46.3%)	69 (41.1%)
	>5	23 (10.4%)	5 (9.3%)	18 (10.7%)
	No data	11 (5.0%)	4 (7.4%)	7 (4.2%)
Type of residence	Rural	176 (79.3%)	43 (79.6%)	133 (79.2%)
	Urban	46 (20.7%)	11 (20.4%)	35 (20.8%)
Household source of income	Child support grant	140 (63.1%)	28 (51.9%)	112 (66.7%)
	Salary	10 (4.5%)	2 (3.7%)	8 (4.8%)
	No source of income	20 (9.0%)	8 (14.8%)	12 (7.1%)
	Both salary and grant	52 (23.4%)	16 (29.6%)	36 (21.4%)
Health systems and programmes				
Supplements received	Yes	173 (77.9%)	41 (75.9%)	132 (78.6%)
	No	49 (22.1%)	13 (24.1%)	36 (21.4%)
Nutrition education given	Yes	203 (91.4%)	45 (83.3%)	158 (94.0%)
	No	19 (8.6%)	9 (16.7%)	10 (6.0%)
Follow-up date given	Yes	175 (78.8%)	40 (74.1%)	135 (80.4%)
	No	47 (21.2%)	14 (25.9%)	33 (19.6%)
Follow-up according to documented schedule	Yes	140 (63.1%)	28 (51.9%)	112 (66.7%)
	No	82 (36.9%)	26 (48.1%)	56 (33.3%)

Variables are presented as frequencies (N) and percentages. Percentages in the columns for total population, documented readmission, and no documented readmission were based on the full sample of 222 records; documented readmissions, n = 54; and no documented readmission cases, n = 168. Missing information is presented as “no data”. Documented readmission refers to a subsequent admission for undernutrition at the Bela-Bela District Hospital. The associations between categorical variables and documented readmission were assessed using chi-squared test. A *p*-value of 0.05 was considered statistically significant. WHZ: weight-for-height Z-score; MUAC: mid-upper arm circumference.

**Table 2:** Multivariable logistic regression of factors associated with documented readmission for undernutrition among children under 5 years of age.

Predictor	Comparison	AOR	95% CI	*p*-Value	Interpretation
History of undernutrition admission	Yes vs. No	4.12	2.00–8.50	<0.001	Strongest adjusted association
Nutritional status	Severe vs. Moderate	1.80	0.90–4.50	0.072	Not statistically significant
Oedema	Any vs. None	1.20	0.60–3.00	0.500	Not statistically significant
Caregiver employment	Unemployed vs. Employed	1.80	0.80–5.00	0.120	Not statistically significant
Documented nutrition education	No vs. Yes	3.10	1.40–6.90	0.005	Significant adjusted association
Follow-up date documented	No vs. Yes	2.20	1.05–4.60	0.036	Significant adjusted association
Documented attendance according to schedule	No Documented Attendance According to Schedule vs. Documented Attendance According to Schedule	1.95	1.05–3.60	0.030	Significant adjusted association
Caregiver education	Lower vs. Secondary/Tertiary	1.70	0.98–2.95	0.058	Borderline; not significant at 0.05
Model information	Complete-Case, N = 206; Events = 49; Non-Events = 157; 8 Predictor Parameters; ≈6.1 Events/Predictor				

AOR: adjusted odds ratio; CI: confidence interval; WHZ: weight-for-height Z-score; MUAC: mid-upper arm circumference. Multivariable regression was used to identify factors independently associated with documented readmission. Categories were collapsed where necessary to support model stability. A *p*-value below 0.05 was considered statistically significant. The total case analysis included 206 children with available data, comprising 49 with documented readmission and 157 without documented readmission. Caregiver education was categorised as lower education (no formal education or primary education) and secondary (secondary or higher).

## Data Availability

Data are available upon request from the corresponding author due to the University of Pretoria’s policy on data publication.
